# Maternal Low-Level Lead Exposure and Fetal Growth

**DOI:** 10.1289/ehp.0901561

**Published:** 2010-06-21

**Authors:** Motao Zhu, Edward F. Fitzgerald, Kitty H. Gelberg, Shao Lin, Charlotte M. Druschel

**Affiliations:** 1 Department of Community Medicine, West Virginia University, Morgantown, West Virginia, USA; 2 Department of Epidemiology and Biostatistics, State University of New York at Albany, Rensselaer, New York, USA; 3 Bureau of Occupational Health, New York State Department of Health, Troy, New York, USA; 4 Bureau of Environmental and Occupational Epidemiology, New York State Department of Health, Troy, New York, USA

**Keywords:** birth weight, blood lead, epidemiology, fetal growth, low-level lead exposure, pregnancy, preterm birth, small for gestational age

## Abstract

**Background:**

Limited epidemiologic studies have examined the association between maternal low-level lead exposure [blood lead (PbB) < 10 μg/dL] and fetal growth.

**Objective:**

We examined whether maternal low-level lead exposure is associated with decreased fetal growth.

**Methods:**

We linked New York State Heavy Metals Registry records of women who had PbB measurements with birth certificates to identify 43,288 mother–infant pairs in upstate New York in a retrospective cohort study from 2003 through 2005. We used multiple linear regression with fractional polynomials and logistic regression to relate birth weight, preterm delivery, and small for gestational age to PbB levels, adjusting for potential confounders. We used a closed-test procedure to identify the best fractional polynomials for PbB among 44 combinations.

**Results:**

We found a statistically significant association between PbB (square root transformed) and birth weight. Relative to 0 μg/dL, PbBs of 5 and 10 μg/dL were associated with an average of 61-g and 87-g decrease in birth weight, respectively. The adjusted odds ratio for PbBs between 3.1 and 9.9 μg/dL (highest quartile) was 1.04 [95% confidence interval (CI), 0.89–1.22] for preterm delivery and 1.07 (95% CI, 0.93–1.23) for small for gestational age, relative to PbBs ≤ 1 μg/dL (lowest quartile). No clear dose–response trends were evident when all of the quartiles were assessed.

**Conclusions:**

Low-level PbB was associated with a small risk of decreased birth weight with a supralinear dose–response relationship, but was not related to preterm birth or small for gestational age. The results have important implications regarding maternal PbB.

With the banning of lead-based paint in 1977, and the phasing out of lead-based gasoline in the 1980s and its ban in 1996, the blood lead (PbB) concentration among the general U.S. population has been declining steadily [Centers for Disease Control and Prevention (CDC) 2005]. However, the general population exposure to low lead levels continues because of the widespread use of lead and its ubiquitous nature (CDC 2005). According to the 2003–2004 National Health and Nutrition Examination Survey (CDC 2005), the mean PbB among women 18–49 years of age was 1.2 μg/dL, with a 95th percentile of 2.6 μg/dL.

PbBs < 10 μg/dL induce adverse effects in humans, including elevated blood pressure, impaired nervous system development, delayed sexual maturation, neurobehavioral effects, depressed renal glomerular filtration rate, and reduced heme synthesis [Agency for Toxic Substances and Disease Registry (ATSDR) 2007]. Furthermore, a clear threshold for these sensitive effects has not been identified (ATSDR 2007). Maternal lead can readily cross the placenta and enter fetal blood circulation starting around week 12–14 of pregnancy, making the fetus susceptible to lead poisoning (Lin et al. 1998).

It is biologically plausible that lead can induce low birth weight, preterm birth, and small for gestational age. Lead can potentially impair normal fetal bone growth by competing with calcium for deposition into bone because lead and calcium have similar chemical characteristics (Potula 2005). Experimental evidence provides support for a potential effect of lead on preterm birth. Lead impedes collagen synthesis and praline hydroxylation in mouse, which may have deleterious effects on chorioamniotic membrane structure and induce its premature rupture (Torres-Sanchez et al. 1999). Rats exposed to lead have reduced bone calcium content, reduced trabecular bone volume, altered growth plate morphology, and enhanced activities of spontaneous uterine contraction (Irgens 1998; Torres-Sanchez et al. 1999).

Limited epidemiologic studies have been conducted to examine maternal low-level lead exposure and fetal growth, especially using PbBs (Irgens 1998; Magri et al. 2003; Rothenberg et al. 2002; Sowers 2002; Torres-Sanchez et al. 1999). Some studies included both low-level and high-level lead exposures, restricting the conclusions regarding low-level lead exposure alone (Torres-Sanchez et al. 1999). Other studies are based on convenience samples such as prenatal clinic and Medicaid participants, limiting their generalizability (Sowers 2002).

Our study was designed to help address some of these issues, using a large population-based PbB registry in New York state. The objectives were to examine whether maternal low-level PbB exposure (< 10 μg/dL) was inversely associated with birth weight and directly associated with the risk of preterm birth, and small for gestational age.

## Methods

### Study population and data sources

The study population comprised upstate New York (New York State, excluding New York City) mothers 15–49 years of age from 2003 through 2005 who had a PbB test before or at the delivery date, and their singleton live births. PbBs were obtained from the New York State Heavy Metals Registry (HMR), which has maintained a statewide database since 1982 and receives reports on exposure to heavy metals, including lead, mercury, arsenic, and cadmium, from physicians and laboratories (New York State Department of Health Bureau of Occupational Health 2008a). In 1992 the reporting requirement was changed from 25 μg/dL to include all test reports regardless of level (New York State Department of Health Bureau of Occupational Health 2008a). Information on birth outcomes and potential confounders was acquired from the birth certificate files, which are maintained by the New York State Department of Health, Bureau of Biometrics.

### Study design and data linkage

A retrospective cohort design was used. The existing HMR records were linked with birth certificate files to form the study base. At first, women with multiple PbB reports were identified through deterministic matching techniques and transposed into one record containing information on all reporting dates and PbBs. To minimize the issues of data entry errors or missing values on identifiers, 10 deterministic identifiers were created using components from variables including the case number, social security number, date of birth, first name, last name, telephone number, ZIP code, street address, sex, and street address of the provider or physician ordering test. During each step, 50 matches were randomly selected and reviewed to ensure that the matches were accurate, using all the potential identifying variables: first name, last name, middle name, date of birth, street address, ZIP code, city, state, phone number, sex, street address, name of the provider or physician ordering test, and reporting laboratory identification number. A total of 215,426 women 15–49 years of age were identified from 245,050 PbB tests that reported < 10 μg/dL from 2003 through 2005.

PbB data were then matched with birth certificates to identify women who delivered live infants. Twenty deterministic identifiers were created using components from variables including date of birth, social security number, first name, middle name, last name, phone number, residential street, and ZIP code of the mother, and residential street and ZIP code of the father. A total of 44,932 singleton live births were identified with at least one PbB test by delivery and the maximal lead level < 10 μg/dL. We then excluded records with implausible birth weight–gestational age combinations (Alexander et al. 1996) to reduce the sample size to 44,873. Approximately 3.5% of mothers had multiple singleton births during the 3-year period, and we randomly selected one birth to finalize 43,288 mother–infant pairs. Approximately 3.0% of women received multiple PbB tests, so we similarly selected one test result at random.

This study was approved by the New York State Department of Health and the State University of New York at Albany institutional review boards.

### Study variables

#### Exposure

PbB concentration was obtained from the HMR PbB reports. The study level was restricted to < 10 μg/dL, which accounted for 99.2% of reports. Atomic spectrometry is the method for routine screening and diagnostic work (Parsons 1993). Its accuracy is ± 1 μg/dL and the detection limit is 1 μg/dL (Parsons 1993). Any errors in the measurement of PbB would be expected to be nondifferential according to low birth weight and other fetal growth outcomes. Laboratories are required to pass three of the quarterly proficiency tests every year by the New York State Department of Health, Wadsworth Center for Laboratories and Research, to ensure the accuracy and comparability (Lin et al. 1998). The coefficient of variation was approximately 7% among all laboratories in 2005 (New York State Department of Health Wadsworth Center 2006).

#### Outcomes

Birth outcomes were abstracted from the birth certificate files. Only singleton live births were selected. Birth weight was examined as a continuous variable. Preterm birth was defined as the gestational age < 37 completed weeks from the date of the last menstrual period (March of Dimes Foundation 2007). Small for gestational age was defined as the birth weight below the 10th percentile of birth weight for gestational age based on the distribution of 1996–2000 national birth weight by gestational week from week 25 through week 42 (Boulet et al. 2006). Binary low birth weight (< 2,500 g) was not examined in multiple variable analysis because continuous birth weight provides more statistical power to detect subtle effects. In addition, low birth weight is a mix of preterm, growth-restricted, and constitutionally small births; preterm birth and small for gestational age were examined in this study. Regarding the accuracy of outcomes recorded in New York State birth certificates, the dates of last menses reported in the birth certificate exactly agreed with those recorded in medical records for 87% (Roohan et al. 2003). The agreement rate was increased to 93% when the tolerance was 1 week (Roohan et al. 2003).

### Confounders

In addition to the timing of lead test in relation to the date of delivery, various potential confounders were abstracted from the BC files: maternal race (Caucasian, African American, other); maternal ethnicity (Hispanic or not); maternal age at the time of delivery; maternal education (less than high school graduate, high school graduate, some college or college degree, graduate education); participation in financial assistance programs (e.g., Medicaid; Family Health Plus; Women, Infants, and Children; other) (yes or no); self-reported maternal smoking during pregnancy (yes or no); self-reported maternal alcohol consumption during pregnancy (yes or no); self-reported illicit drug use during pregnancy (yes or no); trimester when prenatal care began (first trimester, second trimester, third trimester, or no prenatal care); parity (zero, one, two or more previous live births); sex of child; in wedlock (yes or no); and prepregnancy body mass index.

### Statistical analysis

For continuous outcomes (birth weight in grams and gestational age in days), we fitted multiple linear regression with fractional polynomials (Royston et al. 1999). We explored one or two terms of fractional polynomials in term of x^p^ for PbB, where the power p is from −2, −1, −0.5, 1, 2, 3, and natural logarithmic transformation. The selection of final fractional polynomials was based on a closed-test procedure, which maintains the overall type 1 error (alpha level) of 0.05 for tests among 44 different combinations (Royston et al. 1999). For each outcome, a subset of biologically plausible risk factors in addition to PbB was selected to enter the model as potential confounders; those that remained with a significance level of 0.2 were retained (Dales and Urg 1978; Mickey and Greenland 1989; Royston et al. 1999). Fractional polynomials were assessed for continuous confounders including gestational age and maternal age for birth weight outcome. Because the limit of detection for the routine screening and diagnostic laboratory method is 1 μg/dL (Parsons 1993), we conducted sensitivity analysis by *a*) comparing all records; *b*) excluding PbBs of 0 μg/dL; *c*) excluding PbBs < 1 μg/dL.

Furthermore, the quartiles of PbBs (≤ 1 μg/dL; > 1 μg/dL to 2 μg/dL; > 2 μg/dL to 3 μg/dL; > 3 μg/dL to < 10 μg/dL) were used for binary outcomes including preterm birth and small for gestational age. Adjusted odds ratios (aORs) of PbBs were estimated from logistic regression with fractional polynomials (Allison 1999; Royston et al. 1999). The quartiles of PbBs were forced into the model. A closed-test procedure was used to identify the 1 of 44 combinations of one or two fractional polynomials with the best model fit for continuous confounder: maternal age. The criteria for selecting and retaining confounders in the logistic regression were similar to those for linear regression. Analyses were conducted using STATA version 11 (StataCorp, College Station, TX, USA).

## Results

The average PbB concentration was 2.1 μg/dL, and the median was 2 μg/dL (Table 1). The average number of days from lead test to delivery was 203, and the 90th percentile was 237. Most PbB tests were conducted between the date of last menses and the date of delivery. The average birth weight was 3,331 g, and gestational age was 38.8 weeks. Table 2 presents the distribution of selected categorical maternal and infant characteristics. Approximately 68% of births were to white women, and Hispanics accounted for 20%. The rates of low birth weight, preterm birth, and small for gestational age were 6.3%, 8.1%, and 9.5%, respectively.

**Figure 1:**
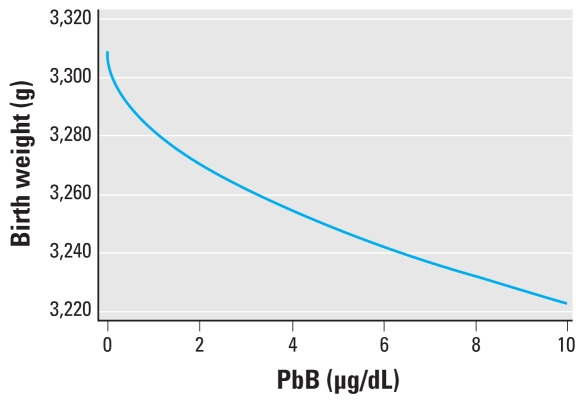
Model-based dose–response relationship.

A model that assumed a linear relation between the square root of PbB and birth weight fit the data better than models with all other combinations of fractional polynomial terms evaluated. Consequently, our final model included only a single term for PbB (raised to the 0.5 power), with an adjusted coefficient of −27.4 [95% confidence interval (CI), −37.7 to −17.1]. Estimated changes in birth weight with a 1-μg/dL change in PbB varied across the PbB distribution in the study population, consistent with the supralinear shape of the dose–response curve dictated by the model, so that a 1-μg/dL change in PbB from 0 μg/dL to 1 μg/dL was associated with a 27.4-g decrease in mean birth weight, whereas a 1-μg/dL change in PbB from 9 μg/dL to 10 μg/dL was associated with a 4.4-g decrease in mean birth weight (from a predicted mean decrease relative to predicted mean birth weight when PbB = 0 of 82.3 g to 86.7 g) (Table 3). Therefore, the model predicts the strongest estimated effects at the lowest levels of exposure, without a lower threshold of PbB below which there would be no predicted effect on birth weight. Figure 1 displays this dose–response relationship.

As for sensitivity analysis, the best-fit fractional polynomials were PbB^−1^ + PbB^−1^ × logarithmic-transformed PbB, after excluding PbB of 0 μg/dL from analysis (data not shown). Compared with PbB of 0.5 μg/dL, PbB of 9.5 μg/dL was associated with a 51-g decrease in birth weight. Compared with PbB of 1 μg/dL, PbB of 10 μg/dL was associated with a 32-g decrease in birth weight. When PbBs < 1 μg/dL were excluded, untransformed PbB fit the data the best, and the linear regression coefficient was a 7.0-g decrease in birth weight for a 1-μg/dL increase in PbB. Therefore, PbB of 10 μg/dL was associated with a 63-g decrease in birth weight, relative to PbB of 1 μg/dL. In contrast, the analysis using all PbBs including zeros and < 1 μg/dL suggested that PbB of 9.5 μg/dL was associated with an 84-g decrease in birth weight, relative to PbB of 0.5 μg/dL, and that PbB of 10 μg/dL was associated with a 59-g decrease in birth weight, relative to PbB of 1 μg/dL. The analysis with all PbBs provided robust estimated effects of lead on birth weight.

A model that assumed a linear relation between untransformed PbB and gestational age in days fit the data better than models with all other combinations of fractional polynomial terms evaluated. Consequently, our final model included only a single linear term for PbB, with an adjusted coefficient of −0.09 (95% CI, −0.24 to 0.05) after adjustment for timing of lead test, maternal age, race, smoking, alcohol consumption, participation in special financial assistance program, parity, and infant sex (data not shown).

Table 4 presents the association between the quartile PbBs and dichotomous outcomes: preterm birth and small for gestational age. There were not clear dose–response trends when all quartiles were assessed. The aORs for PbBs between 3.1 and 9.9 μg/dL (highest quartile) was 1.04 (95% CI, 0.89–1.22) for preterm birth and 1.07 (95% CI, 0.93–1.23) for small for gestational age, relative to ≤ 1 μg/dL (lowest quartile).

## Discussion

Overall, maternal PbBs < 10 μg/dL were associated with a small but statistically significant decrease in birth weight. The decrease in birth weight for a 1-μg/dL increase in PbB ranged from an estimated means value of 4 g (from 9 to 10 μg/dL) to 27 g (from 0 to 1 μg/dL). This is consistent with the estimate of a 6.2-g decrease in birth weight per 1-μg/dL increase in PbB from a study of 272 mother–infant pairs in Mexico (Gonzalez-Cossio et al. 1997); 3.0 g in a study of 4,354 pregnancies in Boston (Bellinger et al. 1991); 0.8 g from a study of 54 term neonates in Turkey (Atabek et al. 2007); and 0.3 g in a study of 55 newborns in Brazil (Zentner et al. 2006), despite that fact that their mean lead levels were higher than ours.

We found that a model of birth weight as a function of square root–transformed PbB provided the best fit to the data. This model predicted estimated effects of lead that were greater at the lower end of the PbB distribution than at higher levels (supralinear dose–response relationship). A similar supralinear relationship has been reported for PbB < 10 μg/dL with IQ and Mental Development Index (Canfield et al. 2003; Lanphear et al. 2005; Tellez-Rojo et al. 2006). A pooled analysis of seven international prospective cohort studies found that the decrease in full-scale IQ score per 1-μg/dL increase in PbB estimated from the linear regression with untransformed PbB was greater among children with a maximum PbB < 7.5 μg/dL than in those with a maximum PbB ≥ 7.5 μg/dL (Lanphear et al. 2005). Further analysis suggested a linear relationship between the logarithmic-transformed PbB and IQ (coefficient: 6.9) (Lanphear et al. 2005). An analysis of 294 children found a logarithmic-transformed PbB was linearly associated with Mental Development Index (Tellez-Rojo et al. 2006). The estimated effect of lead estimated from the linear regression with untransformed PbB was larger at < 5 μg/dL than between 5 and 10 μg/dL (Tellez-Rojo et al. 2006). Researchers have used quadratic term (Canfield et al. 2003) and logarithmic transformations (Lanphear et al. 2005; Tellez-Rojo et al. 2006) to describe the supralinear relationship between lead and intellectual impairment. We found that the square root transformation provided the best fit for birth weight, compared with 43 other fractional polynomials linear, reciprocal, logarithmic, square foot, quadratic, and cubic terms. Further studies are needed to confirm whether the supralinear relationship between PbB and birth weight is best described with a square root transformation. Consistent with previous studies of intellectual development (Canfield et al. 2003; Lanphear et al. 2005; Tellez-Rojo et al. 2006), our analysis supports that there is no clear threshold for the effects of lead on sensitive outcomes such as birth weight.

Bellinger et al. reported that the mean gestational age was 0.3 week longer among those with umbilical cord PbBs 5.0–9.9 μg/dL, relative to PbBs < 5.0 μg/dL (Bellinger et al. 1991). In contrast, we found that a 1-μg/dL increase in maternal PbB was associated with a statistically nonsignificant 0.09-day decrease in gestational age. Similarly, Jelliffe-Pawlowski et al. reported that among women with PbB ≥ 10 μg/dL, a 1-μg/dL increase in lead level was associated with an average 0.3-day decrease in gestational age (Jelliffe-Pawlowski et al. 2006). In a case–control study of 620 pregnant women in Mexico City, compared with umbilical cord PbBs < 5.1 μg/dL, the aOR of preterm birth for lead level 5.1–9.0 μg/dL was 2.72 (95% CI, 1.03–7.19) among primiparous women, but 0.48 (95% CI, 0.21–1.08) among multiparous women (Torres-Sanchez et al. 1999).

Bellinger et al. found that lead levels between 5 and 9.9 μg/dL were not statistically related to increased risk in dichotomous preterm birth and small for gestational age, compared with lead levels < 5 μg/dL (Bellinger et al. 1991). A cohort study by Sowers (2002) of 705 pregnant women in Camden, New Jersey, did not find any statistically significant association with dichotomous preterm birth, or small for gestational age. Consistent with their study, our study did not find statistically significant associations.

Jelliffe-Pawlowski et al. reported that women with PbBs ≥ 10 μg/dL were approximately three times as likely to experience a preterm delivery as women with lead levels < 10 μg/dL (aOR = 3.2; 95% CI, 1.2–7.4) and that their risk of having a small-for-gestational-age infant was more than four times that of women with lead levels < 10 μg/dL (aOR = 4.2; 95% CI, 1.3–13.9) (Jelliffe-Pawlowski et al. 2006). Chen et al. (2006), in a study of 1,611 mother–infant pairs in Taiwan, China, suggested that maternal PbBs of ≥ 10 μg/dL were related to a doubling risk in low birth weight, preterm birth, and small for gestational age compared with maternal PbBs < 10 μg/dL. Highly elevated maternal PbBs would be expected to have adverse effects on fetal growth.

This study has multiple strengths. For example, we used PbBs to measure the absorbed dose circulated in the blood through various exposure routes and sources for pregnant women, which is more accurate than occupation history and other proxy exposure measures. By restricting the lead concentrations to < 10 μg/dL, the associations between maternal lead level and fetal growth found in this study were not influenced by lead concentrations > 10 μg/dL, unlike previous studies that included lead concentrations below and > 10 μg/dL. Because this study was based on a statewide registry and the study lead concentration was close to the lead distribution among the general population, findings should be more generalizable than those based on occupational settings or convenience samples. Furthermore, this study had a large sample size to detect subtle effects.

A possible limitation is selection bias. We found that the mothers in this study were younger and less likely to be Caucasian than other mothers in upstate New York. The linkage rate of PbB reports with birth certificates was higher for mothers 18–19 years of age, African Americans, and with low-weight births, consistent with the selective screening for pregnant women at risk for adverse pregnancy outcome or lead exposure.

Dietary calcium and multiple vitamin use during pregnancy could not be controlled, as they were not collected on either the birth certificates or the HMR. Low dietary calcium intake may increase the gastrointestinal absorption of lead (Bogden et al. 1995). Calcium supplementation may reduce the lead mobilization from bone during pregnancy and therefore reduce the potential lead toxicity (Bellinger 2005; Han et al. 2000). Furthermore, residual confounding may exist because of the potential misclassification or categorization of confounders. For example, maternal smoking was recorded as “yes or no” in birth certificates. Its sensitivity was 89% and specificity was 99% using medical records as a gold standard (Roohan et al. 2003). There was no detailed information on the duration and frequency of smoking.

The results of this study have important implications regarding the recommended action level for childhood PbB. Although 10 μg/dL is the current reference level set by the CDC (ATSDR 2007), this study suggests that maternal PbBs < 10 μg/dL may affect fetal growth. This issue is of public health significance; in 2005, the HMR received about 84,000 reports on women in New York state with PbBs < 10 μg/dL, and most of the reports were regarding women of reproductive age. Our study supports the continuation of lead screening during pregnancy, especially among women who are at risk because of current high-dose exposure, which is recommended by the New York State Department of Health (New York State Department of Health Bureau of Occupational Health 2008b).

## Conclusion

Among pregnant women whose PbB was < 10 μg/dL, PbB (square root transformed) was inversely associated with birth weight. Such findings suggest that the decrease in birth weight per 1-μg/dL increase in PbB was greater at lower concentrations than at higher concentrations without evidence of a lower threshold of effect. These results are important, given the high prevalence of low-level lead exposure among pregnant women and the controversy regarding the recommended action level for maternal PbB.

## Figures and Tables

**Table 1:** Maternal and infant quantitative characteristics, upstate New York, 2003–2005.

			Selected percentiles						
Characteristic	*n*	Mean	Minimum	10th	25th	50th	75th	90th	Maximum
PbB (μg/dL)	43,288	2.1	0	1	1	2	3	3	9.9
Days from lead test to date of birth (day)	43,288	203	0	110	170	204	223	237	1,082
Maternal age (years)	43,288	27.6	15	20	23	27	32	36	49
Body mass index (kg/m^2^)	40,797	26.4	12.5	19.9	21.9	24.9	29.4	35.2	66.5
Gestational age (week)	43,288	38.8	20	37	38	39	40	41	44
Birth weight (g)	43,288	3,331	205	2,680	3,030	3,365	3,686	3,997	5,610

**Table 2:** Maternal and infant qualitative characteristics, upstate New York, 2003–2005 (total *n* = 43,288).

Characteristic	*n*	Percentage^a^
Race		
Caucasian	29,434	68.0
African American	7,113	16.5
Other	6,689	15.5
Missing value	52	

Ethnicity		
Hispanic	8,447	19.7
Missing value	492	

Education		
Less than high school graduate	10,054	23.4
High school graduate	11,675	27.2
Some college or bachelor degree	16,857	39.3
Graduate study	4,337	10.1
Missing value	365	

Smoking		
Yes	8,834	20.5
Missing value	149	

Alcohol drinking		
Yes	493	1.1
Missing value	196	

Drug abuse		
Yes	1,216	2.9
Missing value	973	

Financial assistance program		
Yes	25,803	59.8
Missing value	114	

Start of prenatal care visit		
First trimester	29,187	72.9
Second trimester	8,811	22.0
Third trimester or no prenatal care visit	2,056	5.1
Missing value	3,234	

Parity		
0	17,376	40.4
1	13,715	32.0
2 or more	11,823	27.6
Missing value	374	

In wedlock		
Yes	20,378	47.4
Missing value	261	

Infant sex		
Male	22,154	51.2

Low birth weight		
Yes	2,744	6.3

Preterm birth		
Yes	3,519	8.1

Small for gestational age		
Yes	4,092	9.5
Missing value	112	

a The calculation of percentage excluded missing values. There were no missing values for infant sex, low birth weight, and preterm birth.

**Table 3:** Association between PbB concentration and birth weight, upstate New York, 2003–2005.

PbB concentration (μg/dL)	Difference in birth weight in grams (model based)^a^	
	Estimate	95% CI
0	Reference	
1	−27.4	−17.1 to −37.8
2	−38.8	−24.1 to −53.4
3	−47.5	−29.6 to −65.4
4	−54.8	−34.2 to −75.5
5	−61.3	−38.2 to −84.4
6	−67.2	−41.8 to −92.5
7	−72.5	−45.2 to −99.9
8	−77.6	−48.3 to −106.8
9	−82.3	−51.2 to −113.3
10	−86.7	−54.0 to −119.4

a The model was a linear regression with fractional polynomials after adjustment for timing of lead test, gestational age, maternal age, race, Hispanic ethnicity, education, smoking, alcohol drinking, drug abuse, in wedlock, participation in special financial assistant program, parity, and infant sex. PbB concentration was transformed using a square root. The coefficient was −27.4 with an SE of 5.3.

**Table 4:** Association between maternal PbB level and preterm birth, and small for gestational age, upstate New York, 2003–2005.

	Preterm birth			Small for gestational age		
Maternal PbB level	Cases (*n*)	aOR^a^	95% CI	Cases (*n*)	aOR^b^	95% CI
≤ 1.0	1,069	1.00	Reference	1,168	1.00	Reference
1.1–2.0	1,036	1.03	0.93–1.13	1,268	1.07	0.98–1.17
2.1–3.0	1,171	1.01	0.92–1.10	1,353	1.06	0.98–1.16
3.1–9.9	243	1.04	0.89–1.22	303	1.07	0.93–1.23

a aORs are estimated from logistic regression with fractional polynomials after adjustment for timing of lead test, maternal age at delivery, race, Hispanic ethnicity, smoking, drug abuse, in wedlock, participation in special financial assistance program, parity, and infant sex. The quartiles of PbB concentration were untransformed, and fractional polynomials were used for maternal age.

b aORs are estimated from logistic regression with fractional polynomials after adjustment for timing of lead test, maternal age at delivery, race, education, smoking, drug abuse, in wedlock, participation in special financial assistance program, parity, and infant sex. The quartiles of PbB concentration were untransformed and fractional polynomials were assessed for maternal age.
